# Characterization of the Complete Mitochondrial Genome of *Pleurogenoides japonicus* (Digenea, Pleurogenidae): Comparison With the Members of Microphalloidea and Phylogenetic Implications

**DOI:** 10.1002/ece3.70430

**Published:** 2024-10-16

**Authors:** Jun‐Feng Gao, Tian‐Shuai Ma, Mei‐Ru Hou, Qi An, Xue‐Wei Liu, Xin‐Hui Zhang, Jia‐Wen Wang, Lu Zhou, Xue Wang, Xue Bai, Chen‐Long Jiao, Zhuo Lan, Hong‐Yu Qiu, Chun‐Ren Wang

**Affiliations:** ^1^ Key Laboratory of Bovine Disease Control in Northeast China, Ministry of Agriculture and Rural Affair; Key Laboratory of Prevention and Control of Zoonotic Diseases of Daqing; College of Animal Science and Veterinary Medicine Heilongjiang Bayi Agricultural University Daqing China

**Keywords:** comparison analysis, microphalloidea trematodes, mitochondrial genome, phylogenetic analysis, *Pleurogenoides japonicus*

## Abstract

*Pleurogenoides japonicus* (Trematoda: Microphalloidea) is an important parasite in wood frogs with high infection rates and significant ecological, economic, and societal importance. The scarcity of molecular data for these parasites severely limits population genetics and phylogenetic studies. In the present study, for the first time, we determined and described the entire mitochondrial (mt) genome of *P. japonicus* as the first representative of the family Pleurogenidae. The entire mt genome of *P. japonicus* was circular, with 15,043 bp (GenBank accession number OR900118), containing 36 genes, comprising 12 protein‐coding genes (*cox*1–3, *nad*1–6, *nad*4L, *cyt*b, and *atp*6), two ribosomal RNA genes, 22 transfer RNA genes, and two non‐coding regions. There were 23 intergenic spacers, ranging from 2 to 162 bp, and only one 40 bp overlap between *nad*4L and *nad*4 genes in the *P. japonicus* mt genome. The nucleotide composition of *P. japonicus* mt genome exhibited a strong AT bias with a 63.75% A + T content, while the AT‐ and GC‐skews were − 0.435 and 0.407, respectively. Comparative analysis demonstrated that the *P. japonicus* mt genome shared the most common characteristics with Microphalloidea trematodes, and the *cox*1 gene was the longest and most conserved gene in Microphalloidea trematodes. The gene arrangements of Xiphidiata trematodes were of the same order based on protein‐coding genes and rRNA genes, except for tRNA. More than two gene arrangement types exist in Echinostomata and Xiphidiata, and the gene rearrangement events mainly occurred in “trnE‐trnG” and “trnG‐trnE”. Phylogenetic analysis suggested that trematodes of the family Pleurogenidae clustered more with Prosthogonimidae than Eucotylidae. The mt genome data of *P. japonicus* provide an accurate genetic marker for further studies of Xiphidiata trematodes.

## Graphical abstract

We determined the complete mitochondrial genome of *Pleurogenoides japonicus*, being the first described of the family Pleurogenidae. The gene arrangement types of Xiphidiata trematodes are diverse, and the gene arrangement events mainly occurred in “trnE‐trnG” and “trnG‐trnE”. Our phylogenetic analyses revealed that Pleurogenidae was cluster to Prosthogonimidae than Eucotylidae.

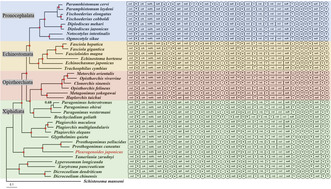

## 1 Introduction

Frogs are among the most important components of biological communities and they play a vital role in connecting water and land environments in ecosystems (Morrison and Hero 2003). They are natural enemies of many pests and are of great significance in the elimination of agricultural pests. Frogs are amphibians and they have a high risk of exposure to pathogens. Many metazoan parasites have frogs as their preferred vertebrate hosts (Hassl 2010). Parasitic diseases are among the most serious factors affecting frog survival. They not only affect the health of frogs but also some zoonotic parasites can even cause human diseases (Eamsobhana 2014).

The genus *Pleurogenoides*, proposed by Travassos in 1921, represents a diverse group of parasitic trematodes that has long attracted the attention of evolutionary ecologists and parasitologists. Species in the genus *Pleurogenoides* require three different hosts to complete their life cycle. The first intermediate hosts are snails and freshwater snails; the second intermediate hosts are dragonfly nymphs, freshwater crabs, and aquatic bugs, and the definitive hosts are frogs. Eggs from adult trematodes are expelled from the definitive host, and miracidia are released from the eggs and enter the first intermediate hosts. Cercariae are asexually produced and are continually shed from first intermediate hosts. Cercariae seek and infect secondary intermediate hosts, where they encyst as metacercariae and remain dormant until ingestion by definitive hosts. Sexual reproduction occurs in the definitive host gut and large amounts of eggs are excreted through the digestive tract of the host (Figure S1). The entire life cycle takes approximately 2 months for completion (Brinesh and Janardanan 2014; Janardanan and Prasadan 1991; Madhavi, Dhanumkumari, and Ratnakumari 1987; Prasadan et al. 2020).

*Pleurogenoides* species are important parasite in the intestine of frogs and common prevalent in China, which can affect the growth in severe cases (Shinad and Prasadan 2018). Saglam and Arikan (2006) reported that the infection rate of *Pleurogenoides spp*. was 1.69% in *Rana ridibunda* in Turkey (Saglam and Arikan 2006). Kuzmin et al. (2020) reported that the infection rate of *Pleurogenoides spp*. was 24.6% in *Pelophylax esculentus* and 26.7% in *Pelophylax ridibundus* in Ukraine (Kuzmin et al. 2020). Shinad and Prasadan (2018) reported that the infection rate of *Pleurogenoides* spp. was 8.99% in *Euphlyctis cyanophlyctis* and 12.5% in *Hoplobatrachus tigerinus* in India (Shinad and Prasadan 2018). Our research found that *Pleurogenoides japonicus* were common trematodes of *Rana amurensis* in Heilongjiang Province, northeastern China. The genus *Pleurogenoides* comprises more than 26 frog species worldwide (Brinesh and Janardanan 2014). However, there are currently 13 *Pleurogenoides* species have been reported in the literature, namely *Pleurogenoides medians*, *Pleurogenoides gastroporus*, *Pleurogenoides euphlycti*, *Pleurogenoides cyanophlycti*, *Pleurogenoides wayanadensis*, *Pleurogenoides malampuzhensis*, *Pleurogenoides neelimae*, *Pleurogenoides orientalis*, *Pleurogenoides stromi*, *Pleurogenoides compactus*, *Pleurogenoides ovatus*, *Pleurogenoides tener*, and *P. japonicus* (Chaudhary et al. 2020; Lee, Choi, and Lee 1976; Olson et al. 2003). However, most studies have focused on the epidemiology and life history of *Pleurogenoides* spp. (Kuzmin et al. 2020; Saglam and Arikan 2006). The scarcity of molecular data for these parasites severely limits population genetics and phylogenetic studies.

The mitochondrial (mt) genome has been shown to be a useful genetic marker, and has been analyzed in parasites of biomedical and veterinary importance. To overcome these limitations of scarcity of molecular data, it is essential to supplement the genetic data currently available in public databases. Thus, the objectives of the present study were to determine the entire mt genome sequence of *P. japonicus*, compare the features of the mt genomes with those of other Microphalloidea species, and assess the phylogenetic relationships between *P. japonicus* and Plagiorchiida trematodes.

## 2 Materials and Methods

### 2.1 Identification of Trematode Specimens

Adult specimens were collected from *Rana dybowskii* intestines naturally infected with *P. japonicus* in Heilongjiang Province, China. This study was approved by the Animal Ethics Committee of the Heilongjiang Bayi Agricultural University (approval number: DWKJXY2022020). The specimens were washed three times in physiological saline, fixed in 75% ethanol, and stored at −20°C until further use. The adult specimens were firstly identified by morphological characteristics according to previous descriptions (Lee, Choi, and Lee 1976), then the specimens were further identified using a molecular method based on the nuclear large subunit ribosomal DNA (lsrDNA). Total genomic DNA was extracted from individual adult specimen using a TIANamp Genomic DNA Kit (TIANGEN Biotech, Beijing, China) according to the manufacturer's instructions. Phylogenetic analyses based on the partial sequences of lsrDNA within superfamily Microphalloidea using Bayesian inference method to determine the trematode species used in this study (Table S1).

### 2.2 mtDNA Genome Sequencing and Assembly

Shotgun sequencing libraries were prepared using the Nextera DNA Sample Prep Kit (Epicenter, Madison, Wisconsin, USA) and the complete mt genome was sequenced using an Illumina (San Diego, CA, USA) NovaSeq sequencing platform (Personal Biotechnology Co., Ltd., Shanghai, China). The raw sequencing data used in this study were deposited in the public repository BioProject (https://www.ncbi.nlm.nih.gov/bioproject) under accession number PRJNA1059768. Clean data without sequencing adapters were assembled using NOVOPlasty version 4.3.1 (Dierckxsens, Mardulyn, and Smits 2017). Moreover, polymerase chain reaction (PCR) was used to further verify the entire mt genome of *P. japonicus* assembly using five primer pairs designed based on conserved regions (Table S2; Figure S2).

PCR was carried out in a total volume of 25 μL, containing 12.5 μL of PrimeSTAR Max Premix (Takara, Dalian, China), 0.5 μL of each primer (50 pmol/μL), 2 μL of DNA template, and 9.5 μL of double‐distilled H_2_O. The reactions were performed in a thermocycler under the following conditions: denaturation at 92°C for 1 min, followed by 30 cycles of denaturation at 92°C for 2 min, then 30 s of annealing at 45°C–60°C, 30 s to 1 min of extension at 72°C, and 10 min of final extension at 72°C. Each amplicon was examined on a 1.5% agarose gel and photographed. The verified amplicons were sequenced by Sangon Biotech Co., Ltd. (Shanghai, China).

### 2.3 mtDNA Genome Sequence Analysis and Gene Annotation

The assembled mt genome of *P. japonicus* was aligned against the mt genomes of *Prosthogonimus pellucidus* (MZ169556), *Prosthogonimus cuneatus* (NC_050918), and *Tamerlania zarudnyi* (MW334947) available in the GenBank database, using DNAStar (version 5.0 (Burland 2000) to identify genetic boundaries. The mt genome of *P. japonicus* was annotated using MITOS (http://mitos.bioinf.uni‐leipzig.de) (Bernt et al. 2013). The protein‐coding genes (PCGs) were refined using the NCBI ORF finder (Rombel et al. 2002), and the putative secondary structures of 22 tRNA genes were predicted using tRNAscan‐SE (http://lowelab.ucsc.edu/tRNAscan‐SE) (Lowe and Eddy 1997). Codon usage for the 12 PCGs of the *P. japonicus* mt genome was calculated using the Codon Usage web server (www.bioinformatics.org/sms2/codon_usage.html) under the invertebrate mt genetic code. The A + T and G + C contents were calculated using DNAStar version 5.0 (Burland 2000). The AT‐ and GC‐skew values were calculated using the equations: AT‐skew = (A−T)/(A + T) and GC‐skew = (G−C)/(G + C) (Perna and Kocher 1995). The mt genome map of *P. japonicus* was drawn using OrganellarGenomeDRAW software (Lohse et al. 2013). The gene arrangements of the *P. japonicus* mt genome and 35 other Plagiorchiida trematode representative species available in the NCBI dataset were linearized at the 5′ ends of their *cox*3 genes in the H‐strand direction.

### 2.4 Comparative Analysis With Other Related Microphalloidea Species

The sequence diversity rate of the *P. japonicus* mt genome with other related Microphalloidea species at the nucleotide and amino acid levels was calculated using MEGA version 11.0 and DNAStar version 5.0 (Burland 2000; Tamura, Stecher, and Kumar 2021). Comparative analyses were conducted among Microphalloidea species, including the families Prosthogonimidae (*P. pellucidus* and *P. cuneatus*), Eucotylidae (*T. zarudnyi*), and Pleurogenidae (*P. japonicus*). Sliding window analysis was performed to assess the nucleotide divergences of 12 PCGs and two ribosomal RNAs between the four Microphalloidea trematodes using DnaSP version 5.0, with a length of 300 bp (in 10 bp overlapping steps) (Librado and Rozas 2009). Nucleotide diversity was plotted against the midpoints of each window.

### 2.5 Phylogenetic Analysis

The phylogenetic relationships among 36 representative members of Xiphidiata from four suborders, including *P. japonicus* isolated in the present study, were determined based on the amino acid sequences of 12 PCGs using *Schistosoma mansoni* as an outgroup (Table S3). The amino acid sequences of the mt genome were aligned using MAFFT version 7 (Katoh and Standley 2013), and then truncated and ambiguously aligned regions were excluded and processed using the program Gblocks Server (http://molevol.cmima.csic.es/castresana/Gblocks_server.html) (Castresana 2000).

Phylogenetic relationships were reconstructed using the Bayesian inference method in MrBayes version 3.2.4 (Ronquist and Huelsenbeck 2003). The best‐fit substitution model for the phylogenetic analysis with amino acid alignment was screened using the ProtTest under the Akaike information criterion, and the JTT + I + G + F model was chosen as the best model for further analysis (Darriba et al. 2011). A Markov chain Monte Carlo algorithm was used with 1000,000 generations and sampled every 1000 generations. The first 25% of the trees were omitted as burn‐in data and generations were added until the average standard deviation of the split frequencies was less than 0.01. Phylogenetic relationships were estimated using the posterior probabilities (PP) for the Bayesian algorithm (Huelsenbeck and Ronquist 2001). Pylograms were drawn using the TreeView program version 1.65 (Page 1996).

## 3 Results And Discussion

### 3.1 Identification of Trematode Specimens

The morphological examination result showed that the trematode body is oval, measured 1.20–1.43 mm × 0.41–0.62 mm in size, the tegument is carpeted with regularly arranged flattened spines. Oral sucker is slightly larger than ventral sucker. Oral sucker is located in the front of the body, and ventral sucker is located in the middle of the body. Two testes are symmetrical and distributed on either side of the ventral sucker. Genital pore is lateral and situated near the left body margin. Cirrus sac is obvious, extends from the front margin of ventral sucker to genital pore. The ovary is spherical and situated in front of the right testicle. Vitellaria is distributed between oral sucker and testes. Uterus is winding mostly in the posterior of the body and filled with eggs (Figure S3). Based on the morphological characteristics, the trematode obtained in the present study was identified as *P. japonicus*.

**FIGURE 1:**
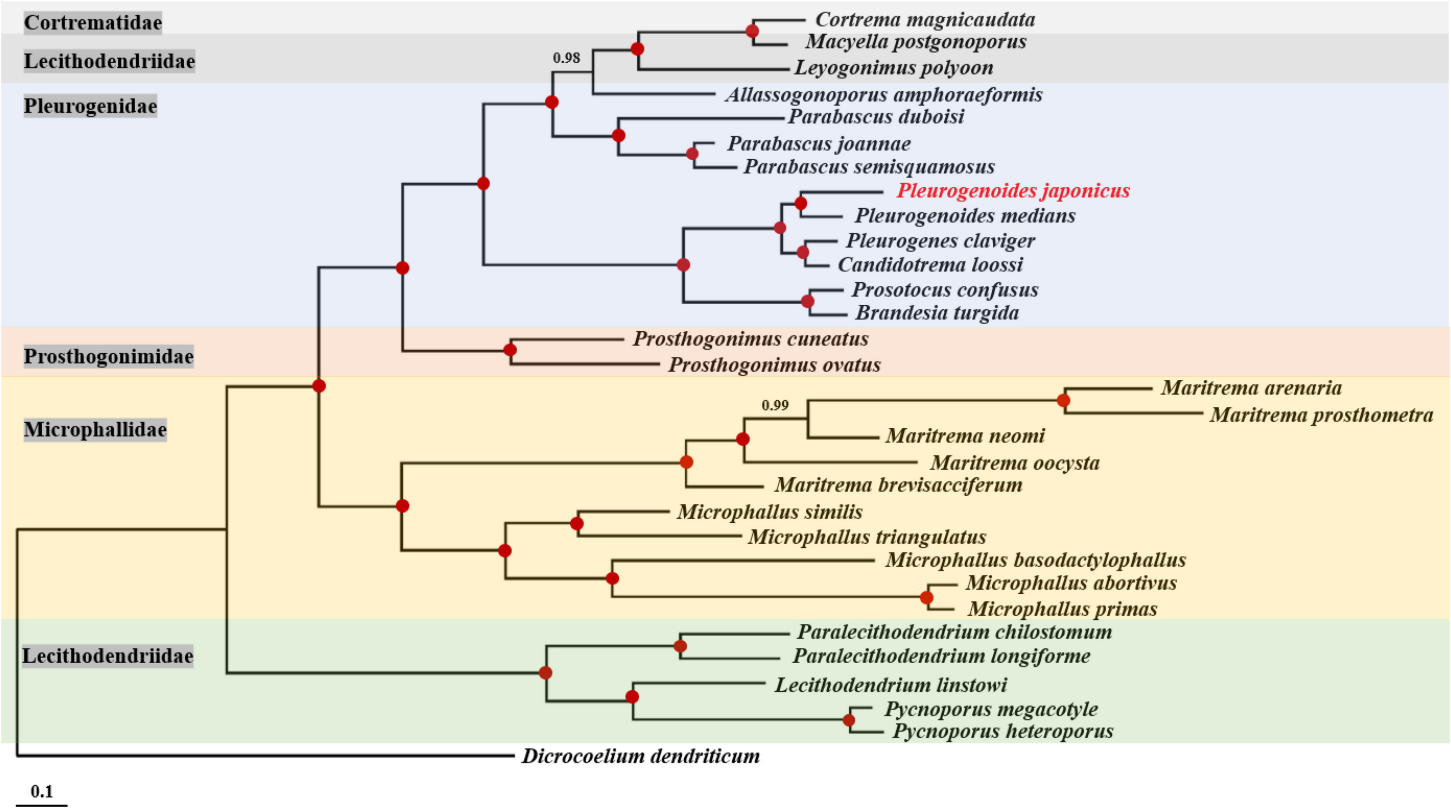
Phylogenetic relationships of *Pleurogenoides japonicus* with 29 representative sequences of superfamily Microphalloidea based on the nuclear large subunit ribosomal DNA sequences analyzed by BI using *Dicrocoelium dendriticum* as the outgroup. Posterior probability values are indicated. Red dots on nodes indicate BPP = 1.00.

The lsrDNA sequence of *P. japonicus* was further assembled from our shotgun sequencing libraries in the present study. The lsrDNA sequence (GenBank accession number PQ285814) showed 98.4% identity with *P. medians* corresponding sequences (GenBank accession number AF433670) available in GenBank. The partial lsrDNA sequence of *P. japonicus* was analyzed together with other 29 representative sequences of superfamily Microphalloidea to assess the phylogenetic relationships, *Dicrocoelium dendriticum* was used as outgroup (Figure 1). Bayesian analysis showed that *P. japonicus* was cluster together with *P. medians*, which both belong to Genus *Pleurogenoides*. The phylogenies within superfamily Microphalloidea based on partial lsrDNA sequences was consistent with previously reported (Chaudhary et al. 2020). Thus, the adult trematode obtained in the present study was identified as *P. japonicus* based on morphological characteristics and molecular data.

### 3.2 General Features of the *P. japonicus* mtDNA Genome

**FIGURE 2:**
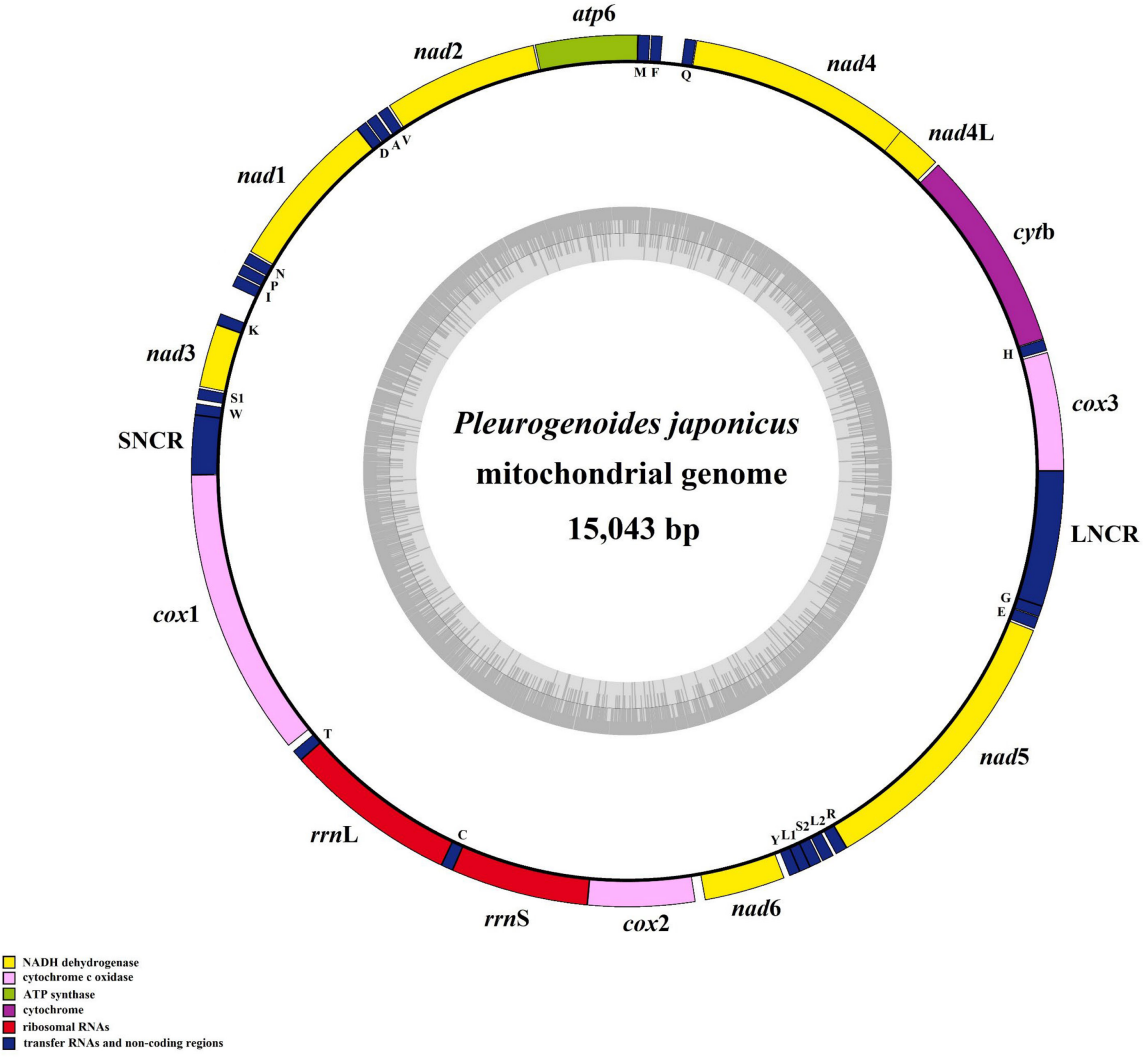
Gene map of the mitochondrial genome of *Pleurogenoides japonicus*. All 22 tRNAs are designated by the one‐letter code for the corresponding amino acid, with numerals differentiating each of the two leucine and serine‐specifying tRNAs (L1 and L2 for codon families CUN and UUR, respectively; S1 and S2 for codon families UCN and AGN, respectively), LNCR and SNCR refers to long and short non‐coding region. All genes are transcribed in the anticlockwise direction.

The complete circular mt genome of *P. japonicus* was 15,043 bp long (GenBank accession number OR900118). The mt genome of *P. japonicus* contained 36 genes that were transcribed in the same direction, comprising 12 PGCs (*cox*1–3, *nad*1–6, *nad*4L, *cyt*b, and *atp*6), two ribosomal RNA genes (*rrn*L and *rrn*S), 22 transfer RNA genes, and two non‐coding regions (NCRs), LNCR and SNCR (Figure 2; Table 1). However, it lacked the *atp*8 gene, which was consistent with other platyhelminthes (An et al. 2022; Guo et al. 2022). There were 23 intergenic spacers, ranging from 2 to 162 bp, and only one 40 bp overlap between *nad*4L and *nad*4, which has been commonly reported in the mt genomes of Plagiorchiida trematodes (Briscoe et al. 2016; Chang et al. 2016; Gacad et al. 2023; Li et al. 2019). The nucleotide composition of the *P. japonicus* mt genome exhibited a strong AT bias. The A + T content was 63.75% (18.00% for A, 45.75% for T), and the G + C content was 36.25% (25.51% for G, 10.74% for C). The AT‐ and GC‐skews of *P. japonicus* entire mt genome were calculated as −0.435 and 0.407, respectively. These results are consistent with those previously reported for Xiphidiata trematodes (Briscoe et al. 2016; Gacad et al. 2023; Liu et al. 2014; Qian et al. 2018).

**TABLE 1:** Features of the mitochondrial genome of *Pleurogenoides japonicus*.

Genes	Location	Length (bp)	Initiation codons	Termination codons	Anticodons	Intergenic spacers (bp)
*cox*3	1–660	660	ATG	TAG		9
*trn*H	670–734	65			GTG	2
*cyt*b	737–1864	1128	ATG	TAG		17
*nad*4L	1882–2142	261	GTG	TAA		−40
*nad*4	2103–3380	1278	GTG	TAG		2
*trn*Q	3383–3445	63			TTG	122
*trn*F	3568–3630	63			GAA	6
*trn*M	3637–3703	67			CAT	0
*atp*6	3704–4273	570	ATG	TAG		7
*nad*2	4281–5144	864	ATG	TAG		10
*trn*V	5155–5218	64			TAC	12
*trn*A	5231–5294	64			TGC	6
*trn*D	5301–5362	62			GTC	68
*nad*1	5363–6259	897	ATG	TAG		9
*trn*N	6269–6333	65			GTT	7
*trn*P	6341–6401	61			TGG	7
*trn*I	6409–6474	66			GAT	162
*trn*K	6637–6701	65			CTT	0
*nad*3	6702–7055	354	GTG	TAG		9
*trn*S1	7065–7127	63			AGC	21
*trn*W	7149–7212	64			TCA	0
SNCR	7213–7542	330				0
*cox*1	7543–9153	1611	ATT	TAA		39
*trn*T	9193–9256	64			TGT	0
*rrn*L	9257–10,226	970				0
*trn*C	10,227–10,293	67			GCA	0
*rrn*S	10,294–11,060	767				0
*cox*2	11,061–11,657	597	ATG	TAG		52
*nad*6	11,710–12,162	453	ATG	TAG		30
*trn*Y	12,193–12,254	62			GTA	0
*trn*L1	12,255–12,316	62			TAG	0
*trn*S2	12,317–12,383	67			TCA	8
*trn*L2	12,392–12,459	68			TAA	15
*trn*R	12,475–12,542	66			TCG	0
*nad*5	12,543–14,147	1605	ATG	TAG		9
*trn*E	14,157–14,223	67			TTC	2
*trn*G	14,226–14,287	62			TCC	0
LNCR	14,288–15,043	756				0

A total of 10,278 bp of 12 concatenated PCGs of *P. japonicus* encoded 3414 amino acids, excluding termination codons. Codon usage analysis showed that three types of initiation codons (ATG, GTG, and ATT) and two types of termination codons (TAG and TAA) were used in the *P. japonicus* mt genome (Table 1). The most frequently used codons of the *P. japonicus* mt genome were Phe (TTT, 11.35%), followed by Val (GTT, 7.88%) and Leu (TTG, 6.66%). The least frequently used codons were Arg (CGC, 0.06%), Thr (ACC, 0.15%), and Leu (CTC, 0.18%) (Table 2). Similar to most Digenea trematodes, the mt genome of *P. japonicus* contained 22 common tRNAs, ranging from 61 to 68 bp in size. Twenty‐two tRNA genes of *P. japonicus* were 1417 bp in length, and the A + T content was 59.92%. The predicted secondary structures of the 22 tRNAs of *P. japonicus* were similar to those of most Xiphidiata trematodes reported to date, except for the *P. westermani* Korean and Indian isolates, which have 23 and 24 tRNAs in the mt genome, respectively (Biswal et al. 2014) (Figure S4). *rrn*L and *rrn*S of *P. japonicus* were located between *trn*T and *trn*C and between *trn*C and *cox*2, respectively. *rrn*L was 970 bp in length with 62.27% A + T content, whereas *rrn*S was 767 bp in length with 61.41% A + T content. The mt genome of *P. japonicus* contained two NCRs. The LNCR and SNCR were located between *trn*G and *cox*3 and between *trn*W and *cox*1, respectively. The LNCR was 756 bp in length with 68.92% A + T content, whereas the SNCR was 330 bp in length with 68.18% A + T content.

**TABLE 2:** The codon usage for 12 PCGs of *Pleurogenoides japonicus* mitochondrial genome.

Codon	Number	/1000	Fraction	Codon	Number	/1000	Fraction
GCG(Ala)	18	5.25	0.16	CCG(Pro)	9	2.63	0.11
GCA(Ala)	13	3.79	0.11	CCA(Pro)	12	3.50	0.15
GCU(Ala)	68	19.85	0.59	CCU(Pro)	52	15.18	0.64
GCC(Ala)	16	4.67	0.14	CCC(Pro)	8	2.34	0.10
UGU(Cys)	125	36.49	0.87	CAG(Gln)	18	5.25	0.69
UGC(Cys)	18	5.25	0.13	CAA(Gln)	8	2.34	0.31
GAU(Asp)	61	17.81	0.88	CGG(Arg)	13	3.79	0.16
GAC(Asp)	8	2.34	0.12	CGA(Arg)	11	3.21	0.13
GAG(Glu)	64	18.68	0.88	CGU(Arg)	57	16.64	0.69
GAA(Glu)	9	2.63	0.12	CGC(Arg)	2	0.58	0.02
UUU(Phe)	389	113.54	0.96	AGG(Ser)	51	14.89	0.14
UUC(Phe)	17	4.96	0.04	AGA(Ser)	27	7.88	0.07
GGG(Gly)	85	24.81	0.29	AGU(Ser)	79	23.06	0.22
GGA(Gly)	21	6.13	0.07	AGC(Ser)	9	2.63	0.02
GGU(Gly)	156	45.53	0.53	UCG(Ser)	21	6.13	0.06
GGC(Gly)	34	9.92	0.11	UCA(Ser)	21	6.13	0.06
CAU(His)	42	12.26	0.78	UCU(Ser)	144	42.03	0.39
CAC(His)	12	3.50	0.22	UCC(Ser)	15	4.38	0.04
AUU(Ile)	109	31.82	0.87	ACG(Thr)	12	3.50	0.19
AUC(Ile)	17	4.96	0.13	ACA(Thr)	11	3.21	0.18
AAG(Lys)	49	14.30	0.64	ACU(Thr)	34	9.92	0.55
AAA(Lys)	27	7.88	0.36	ACC(Thr)	5	1.46	0.08
UUG(Leu)	228	66.55	0.45	GUG(Val)	108	31.52	0.25
UUA(Leu)	175	51.08	0.34	GUA(Val)	33	9.63	0.08
CUG(Leu)	14	4.09	0.03	GUU(Val)	270	78.81	0.63
CUA(Leu)	10	2.92	0.02	GUC(Val)	17	4.96	0.04
CUU(Leu)	78	22.77	0.15	UGG(Trp)	66	19.26	0.61
CUC(Leu)	6	1.75	0.01	UGA(Trp)	42	12.26	0.39
AUG(Met)	106	30.94	0.65	UAU(Tyr)	154	44.95	0.86
AUA(Met)	58	16.93	0.35	UAC(Tyr)	26	7.59	0.14
AAU(Asn)	37	10.80	0.80	UAG(*)	10	2.92	0.83
AAC(Asn)	9	2.63	0.20	UAA(*)	2	0.58	0.17

### 3.3 Comparative Analysis With Other Related Microphalloidea Species

**FIGURE 3:**
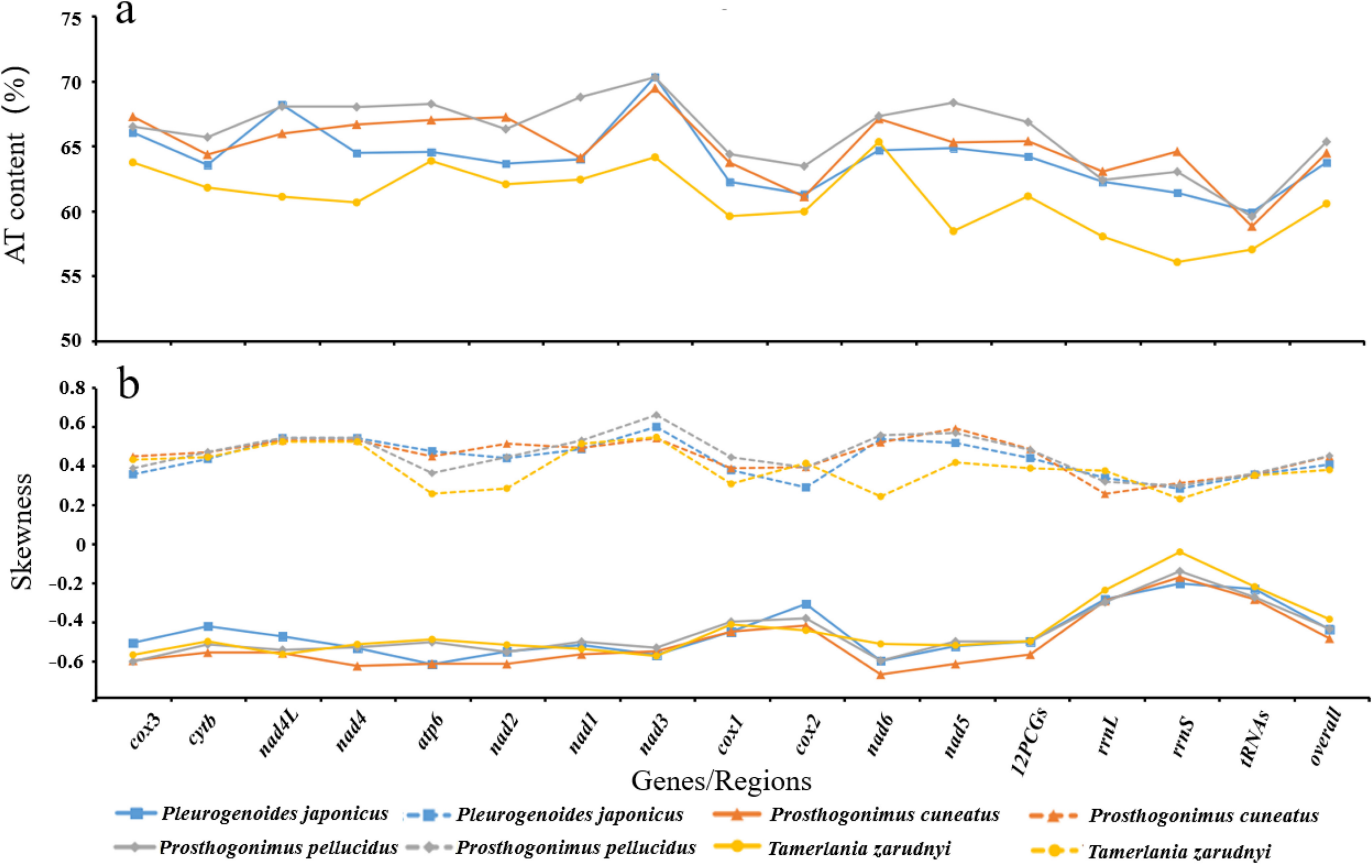
A + T contents and nucleotide skew of genes, individual elements, and the complete mitochondrial genome of four Microphalloidea trematodes. Lines color and point represent a specific Microphalloidea species.

The mt genome sequence of *P. japonicus* (15,043 bp) was similar to that of *P. pellucidus* (15,013 bp), slightly longer than that of *P. cuneatus* (14,829 bp), and shorter than that of *T. zarudnyi* (16,188 bp). *P. japonicus* and *T. zarudnyi* had two NCRs, which was consistent with Gorgoderoidea (*Dicrocoelium chinensis*, *D. dendriticum*, *Eurytrema pancreaticum*, *Lyperosomum longicauda*) and Allocreadioidea (*Brachycladium goliath*) trematodes, but inconsistent with *P. pellucidus* and *P. cuneatus*, which have only one (Suleman Muhammad et al. 2021; Guo et al. 2022). Notably, one NCR was located between *trn*E and *cox*3 in Microphalloidea trematodes, and there was also a large gap between *trn*W and *cox*1 in *P. pellucidus* and *P. cuneatus*, which was consistent with the location of the other NCR identified in *P. japonicus* in the present study. Nevertheless, although *T. zarudnyi* has two non‐coding regions, the LNCR was located between *trn*E and *trn*G, which was inconsistent with the location between *trn*W and *cox*1 in *P. japonicus*. The direction of gene transcription in the *P. japonicus* mt genome was the same as that for the mt genomes of all Microphalloidea trematodes. The lengths of the 12 *P. japonicus* 12 PCGs were in the order of *cox*1 *> nad*5 *> nad*4 *> cyt*b *> nad*1 *> nad*2 *> cox*3 *> cox*2 *> atp*6 *> nad*6 *> nad*3 *> nad*4L. This result was consistent with all Microphalloidea trematodes, but was inconsistent with other Xiphidiata trematodes, in which *nad*5 was the longest PCG (Chang et al. 2016; Gacad et al. 2023; Le et al. 2019). The A + T content and nucleotide skew values were calculated for the four superfamilies of Microphalloidea reported to date (Figure 3). The A + T and G + C contents of *P. japonicus* were 63.75% and 36.25%, respectively, which are in accordance with those of other species in the superfamily Microphalloidea (Figure 3a; Table S4). The characteristic A + T bias in the Microphalloidea trematodes mt genomes was also consistent with that of other Xiphidiata trematodes (Li et al. 2022; Liu et al. 2014; Qian et al. 2018). The AT‐skew values of the *P. japonicus* mt genome were between −0.614 (*atp*6) and − 0.202 (*rrn*S) and the GC‐skew values were between 0.284 (*rrn*S) and 0.6 (*nad*3) (Figure 3b; Table S5). The *P. japonicus* mt genome exhibited negative AT skew values and positive GC skew values, similar to those of other Xiphidiata trematodes (Biswal et al. 2014; Briscoe et al. 2016; Suleman Khan et al. 2020).

**FIGURE 4:**
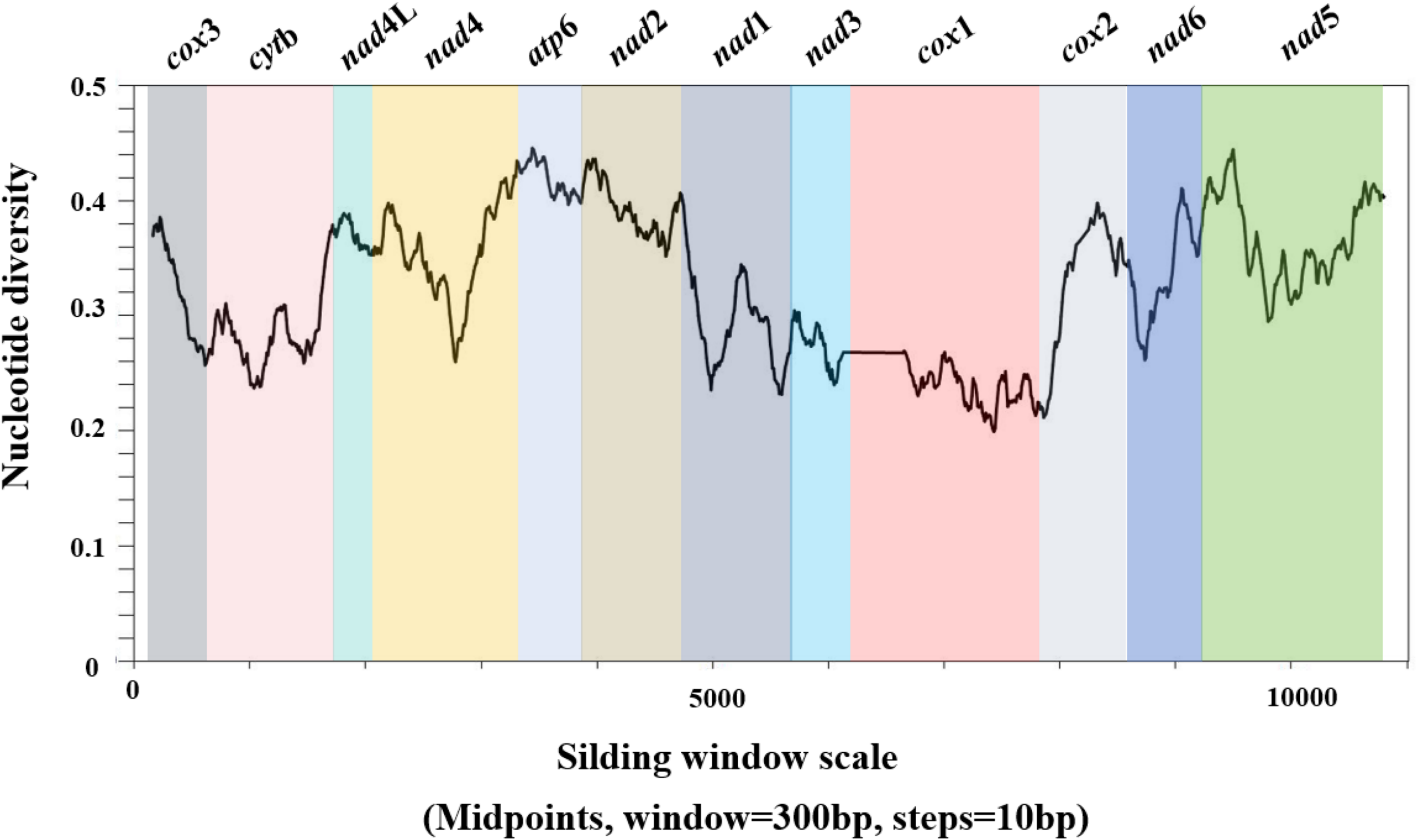
Sliding window analysis of the complete mitochondrial genome sequences of four Microphalloidea trematodes. A sliding window of 300 bp (in 10 bp overlapping steps) was used to estimate nucleotide diversity Pi (*π*) across the alignments. Nucleotide diversity was plotted against the mid‐point positions of each window. Each gene boundary is identified.

Sequence diversity and sliding window analyses were performed to evaluate the diversity of mtDNA genes among the four Microphalloidea trematodes. The nucleotide sequence diversity of the complete mt genomes of the four Microphalloidea trematodes ranged from 16.9% to 41.2%. Likewise, the nucleotide sequence diversity of the 12 PCGs of Microphalloidea trematodes was 18.8%–40.3%, and the amino acid sequence diversity of the 12 PCGs was 18.3%–52.5% (Table 3). Sliding window analysis was then executed by calculating the number of variable positions per 300 bp length of the concatenated nucleotide sequences of the 12 PCGs. The results showed that the highest level of sequence variability was in the *nad*5 gene, and the lowest level of sequence variability was in the *cox*1 gene (Figure 4), indicating that *nad*5 was the least conserved PCG, while *cox*1 was the most conserved PCG in the *P. japonicus* mt genome. This result was consistent with previous studies of *L. longicauda*, *P. cuneatus*, *P. pellucidus*, and *T. zarudnyi*, which found that *cox*1 was the most conserved gene and can be used as a DNA barcode for metazoans (Guo et al. 2022; Mioduchowska et al. 2018; Suleman Khan et al. 2020; Suleman Muhammad et al. 2021).

**TABLE 3:** Nucleotide and/or predicted amino acid (aa) sequence differences for protein‐coding genes among *Pleurogenoides japonicus* (PJ), *Prosthogonimus pellucidus* (PP), *Prosthogonimus cuneatus* (PC) and *Tamerlania zarudnyi* (TZ).

Gene/region	Gene nucleotide length (bp)				Nucleotide difference (%)						Number of aa				aa difference (%)					
	PJ	PP	PC	TZ	PJ/PP	PJ/PC	PJ/TZ	PP/PC	PP/TZ	PC/TZ	PJ	PP	PC	TZ	PJ/PP	PJ/PC	PJ/TZ	PP/PC	PP/TZ	PC/TZ
*atp*6	570	564	561	537	36.8	39.5	49.0	26.5	48.6	47.9	189	187	186	178	59.8	61.0	77.1	33.7	77.3	79.5
*cox*1	1611	1668	1641	2055	24.7	25.3	29.6	17.3	28.1	28.6	536	555	546	684	23.3	23.4	30.5	10.8	28.1	28.9
*cox*2	597	594	594	597	31.1	32.8	36.8	17.5	37.1	38.5	198	197	197	198	38.6	36.5	45.4	11.7	45.9	45.9
*cox*3	660	654	654	651	31.2	29.7	40.0	12.5	41.7	40.9	219	217	217	216	41.4	37.2	60.4	17.1	59.0	59.5
*cyt*b	1128	1125	1122	1113	29.6	27.5	35.8	18.4	34.4	31.3	375	374	373	370	33.4	33.5	44.9	13.9	43.0	41.8
*nad*1	897	900	900	897	26.9	27.3	29.7	18.9	29.2	31.1	298	299	299	298	30.9	30.9	38.6	14.4	38.3	38.9
*nad*2	864	867	867	870	38.2	38.1	50.5	19.4	46.7	44.9	287	288	288	289	54.0	51.4	68.9	26.6	66.3	64.8
*nad*3	354	357	357	357	27.4	23.4	25.6	20.7	37.3	35.9	117	118	118	118	40.2	37.6	51.3	22.0	47.5	44.1
*nad*4	1278	1257	1257	1284	35.4	33.5	41.9	22.0	40.0	39.5	425	418	418	427	45.9	42.8	57.8	24.6	57.8	57.8
*nad*4L	261	288	288	270	34.1	31.8	43.5	20.1	40.7	43.7	86	95	95	89	41.9	38.4	54.7	20.0	53.9	53.9
*nad*5	1605	1596	1596	1596	38.0	38.7	43.9	15.8	43.1	43.1	534	531	531	531	53.9	53.3	60.6	16.2	61.3	61.5
*nad*6	453	456	456	450	34.0	33.8	44.6	24.8	46.5	45.4	150	151	151	149	50.7	52.7	64.9	23.8	70.9	71.6
12 PCGs	10,278	10,326	10,293	10,677	32.8	32.7	40.3	18.8	37.9	39.8	3414	3430	3419	3547	41.5	40.4	52.5	18.3	51.7	51.5
Total	15,043	15,013	14,829	16,188	32.3	31.7	41.2	16.9	40.1	39.8	—	—	—	—	—	—	—	—	—	—

### 3.4 Gene Arrangement and Phylogenetic Analysis

Gene arrangement in mt genome provides a source of information for phylogenetic inference and has been extensively reported previously (Gao et al. 2023; Biswal et al. 2014; Webster and Littlewood 2012). In the present study, the mt genomes of 35 Plagiorchiida trematodes, including *P. japonicus*, were linearized at the 5′ end of their *cox*3 genes in the H‐strand direction. The gene arrangements of all trematodes were in the same order based on the PCGs and rRNA genes (*cox*3—*cyt*b—*nad*4L—*nad*4—*atp*6—*nad*2—*nad*1—*nad*3—*cox*1—*rrn*L—*rrn*S—*cox*2—*nad*6—*nad*5), and the gene rearrangement events only occurred in transposed tRNAs. Conserved gene arrangement was considered as a typical feature of mt genomes (Zhang et al. 2018). It is reported that the gene order among free‐living flatworms differs considerably from the parasitic flatworms. However, the gene order of both free‐living and parasitic flatworms was highly conservative with only a few exceptions in each kind of flatworm (Solà et al. 2015). Our finding was well supported the phenomenon that all trematode in Plagiorchiida trematodes shared an identical gene order based on PCGs and rRNA genes. However, several exceptions have been reported, such as the trematode in genus *Schistosoma*, which occurrence extensive of gene rearrangement events in mt genomes of *Schistosoma spp*. (Webster and Littlewood 2012).

The gene arrangements of Pronocephalata and Opisthorchiata trematodes are conserved, whereas two more gene arrangements exist in Echinostomata and Xiphidiata, indicating that different degrees of trematode evolution exist in Echinostomata and Xiphidiata. The gene arrangement of *P. japonicus* mt genome conforms to the common pattern in comparison to all other sequenced mt genomes, which belonged to type II rearrangement in Plagiorchiida trematodes according to previously reported (Gao et al. 2023). Notably, gene rearrangement events occurred mainly in *trn*E‐*trn*G and *trn*G‐*trn*E mutants. These results are consistent with those of previous reports hypothesizing that the “*trn*E‐*trn*G” gene rearrangement model evolutionarily appeared before the “*trn*G‐*trn*E” gene rearrangement model (Zhang et al. 2019). These gene rearrangements may provide essential clues regarding the evolution and origin of Plagiorchiida trematodes.

**FIGURE 5:**
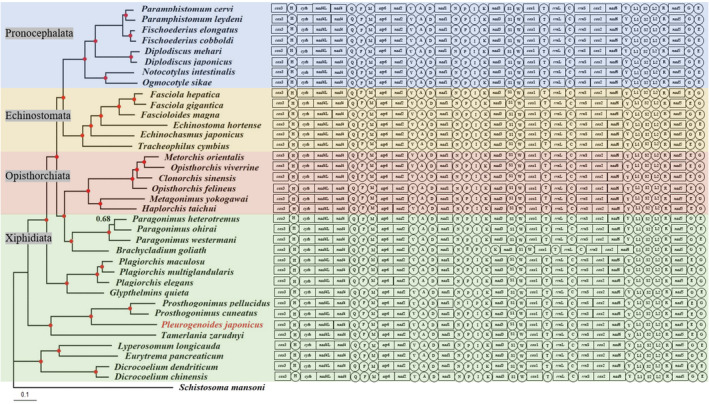
Phylogenetic relationships of *Pleurogenoides japonicus* with other 35 related trmatodes based on the concatenated amino acid sequences of 12 protein coding genes analyzed by BI using *Schistosoma mansoni* as the outgroup. Posterior probability values are indicated. Red dots on nodes indicate BPP = 1.00. The circular mitochondrial genomes were linearized at the 5′ end of *cox*3 gene (except non‐coding regions) for illustration gene arrangement.

Studies on family Pleurogenidae have mainly focused on morphology, life history, and prevalence, and the information of molecular data was rare in the past. For decades, there has been considerable controversy over the taxonomy of Digenea trematodes, especially Plagiorchiida trematodes (Gao et al. 2023; Suleman Muhammad et al. 2021). The mt genome has proved as a valuable genetic marker for systematic studies of trematodes. In this study, the phylogenetic tree was constructed from the concatenated amino acid sequence dataset of the 12 PCGs from 35 Plagiorchiida trematodes using BI approaches (Figure 5).

Two large clades were visibly reflected with high PP and one clade consisting of four representative trematodes of the superfamily Microphalloidea appeared rather basal in the tree, whereas the other clade comprised 32 representative trematodes from 16 families. The suborders Pronocephalata, Echinostomata, and Opisthorchiata were rooted in single branches, whereas the suborder Xiphidiata was not. In the suborder Xiphidiata clade, Paragonimidae (*Paragonimus heterotremus*, *Paragonimus ohirai*, *Paragonimus westermani*) and Brachycladiidae (*B. goliath*) clustered together in the derived suborder Xiphidiata branch, which was closer to the suborder Opisthorchiata than to other suborder Xiphidiata species. Olson et al. (2003) classified the families Paragonimidae and Dicrocoeliidae into the superfamily Gorgoderoidea based on rDNA sequences. Subsequently, Le et al. reported that their dataset did not support the close relationship between Paragonimidae and Dicrocoeliidae within superorder Xiphidiata based on mt genome sequences (Le et al. 2019). Paragonimidae and Brachycladiidae were recently reported to be considerably distinct from other suborder Xiphidiata trematodes (including Dicrocoeliidae) based on their mt genome sequences, which form a distinct branch outside the suborder Xiphidiata branch (Suleman Muhammad et al. 2021). A similar result was also found in the present study, which supported that the Paragonimidae was closer to Brachycladiidae than to Dicrocoeliidae. Phylogenetic analyses also show that the suborder Xiphidiata presented a paraphyletic group in the Plagiorchiida phylogenetic tree based on the mt genome in the present study. Recently, more and more studies have been reported that mt genome phylogenetic analyses using amino acids data have consistently shown suborder Xiphidiata presented a paraphyletic group (Guo et al. 2022; Gao et al. 2023; Atopkin et al. 2023). In the most recently published study, phylogenetic analyses were conducted using 28 species trematodes allocated in suborder Xiphidiata and Opisthorchiata by nucleotide and amino acids sequences. The result also corroborated Xiphidiata as paraphyletic using amino acids sequences, although their dataset was showed Xiphidiata as monophyly using nucleotide sequences (Solórzano‐García et al. 2024). In the superfamily Microphalloidea, the family Prosthogonimidae (*P. pellucidus*, *P. cuneatus*) was clustered with Pleurogenidae (*P. japonicus*) rather than Eucotylidae (*T. zarudnyi*), which was consistent with the previously reported utilizing lsrDNA sequences (Tkach et al. 2003).

We demonstrated that the mt genome can be a novel and useful genetic marker and will enhance our understanding of the systematics and population genetics of Xiphidiata trematodes. However, little information on the mt genome of Xiphidiata trematodes (only one representative species) has been published for the family Pleurogenidae in GenBank. Therefore, the abundance of Xiphidiata trematodes should be explored and more mt genome sequences should be reported to re‐evaluate the phylogenetic relationships between Xiphidiata and other trematodes.

## 4 Conclusions

We determined and described the entire mt genome of *P. japonicus* for the first time in this study, which was the first representative of the family Pleurogenidae to have the mt genome published. Comparative analysis demonstrated that the *P. japonicus* mt genome shares the most common characteristics with those of Microphalloidea trematodes. Phylogenetic analysis suggested that trematodes of the family Pleurogenidae clustered more with Prosthogonimidae than Eucotylidae. The availability of mt genome data for *P. japonicus* provides an accurate molecular marker for further studies of the epidemiology, population genetics, and phylogenetic systematics of Xiphidiata trematodes.

## Author Contributions

**Jun‐Feng Gao:** data curation (equal), funding acquisition (lead), project administration (lead), supervision (equal), writing – review and editing (equal). **Tian‐Shuai Ma:** investigation (equal), methodology (equal). **Mei‐Ru Hou:** data curation (equal), investigation (equal), methodology (equal). **Qi An:** investigation (equal), methodology (equal). **Xue‐Wei Liu:** resources (equal), software (equal). **Xin‐Hui Zhang:** resources (equal), software (equal). **Jia‐Wen Wang:** resources (equal), software (equal). **Lu Zhou:** validation (equal), visualization (equal). **Xue Wang:** validation (equal), visualization (equal). **Xue Bai:** validation (equal), visualization (equal). **Chen‐Long Jiao:** software (equal), validation (equal), visualization (equal). **Zhuo Lan:** writing – original draft (equal). **Hong‐Yu Qiu:** writing – original draft (equal). **Chun‐Ren Wang:** conceptualization (lead), supervision (equal).

## Conflicts of Interest

The authors declare no conflicts of interests.

## Supporting information


**Figure S1.** The life cycle of *Pleurogenoides* trematodes.

**Figure S2.** PCR amplicons from the mitochondrial genome of *Pleurogenoides japonicus*. M: DL 2000 DNA marker, 1: Partial of *nad*5, 2: Partial of *atp*6, 3: Partial of *nad*3, 4: Partial of SNCR, 5: *cox*2; 6: Negative control.

**Figure S3.** Morphological characteristics of *Pleurogenoides japonicus* adult. OS, oral sucker; VS, ventral sucker; OV, ovary; TE, testes; VT, vitellaria; UT, uterus; CS, cirrus sac; GP, genital pore.

**Figure S4.** Predicted structure model of 22 tRNAs from the mitochondrial genome of *Pleurogenoides japonicus*.

**Table S1.** The nuclear large subunit ribosomal DNA sequences information of superfamily Microphalloidea used in phylogenetic analysis.

**Table S2.** Four pairs of primers used for the complete mitochondrial genome assembly validation of *Pleurogenoides japonicus*.

**Table S3.** The complete mitochondrial genome information of trematodes used in phylogenetic analysis.

**Table S4.** The A + T contents of mitochondrial genome in superfamily Microphalloidea trematodes.

**Table S5.** The A + T and G + C skew of mitochondrial genome in superfamily Microphalloidea trematodes.

## Data Availability

The mt genome sequence of *P. japonicus* generated in this study were deposited in the NCBI GenBank under accession number OR900118. The nuclear large subunit ribosomal DNA sequence of *P. japonicus* generated in this study were deposited in the NCBI GenBank under accession number PQ285814. Raw sequencing data used in this study were deposited in the public repository BioProject under accession number PRJNA1059768.
