# Supplementary figures and images for: Characterization of the Complete Mitochondrial Genome of Pleurogenoides japonicus (Digenea, Pleurogenidae): Comparison With the Members of Microphalloidea and Phylogenetic Implications
Source: Ecol Evol. 2024 Oct 16;14(10):e70430. doi: 10.1002/ece3.70430 (PMC11483596; doi:10.1002/ece3.70430)

## Slide 1
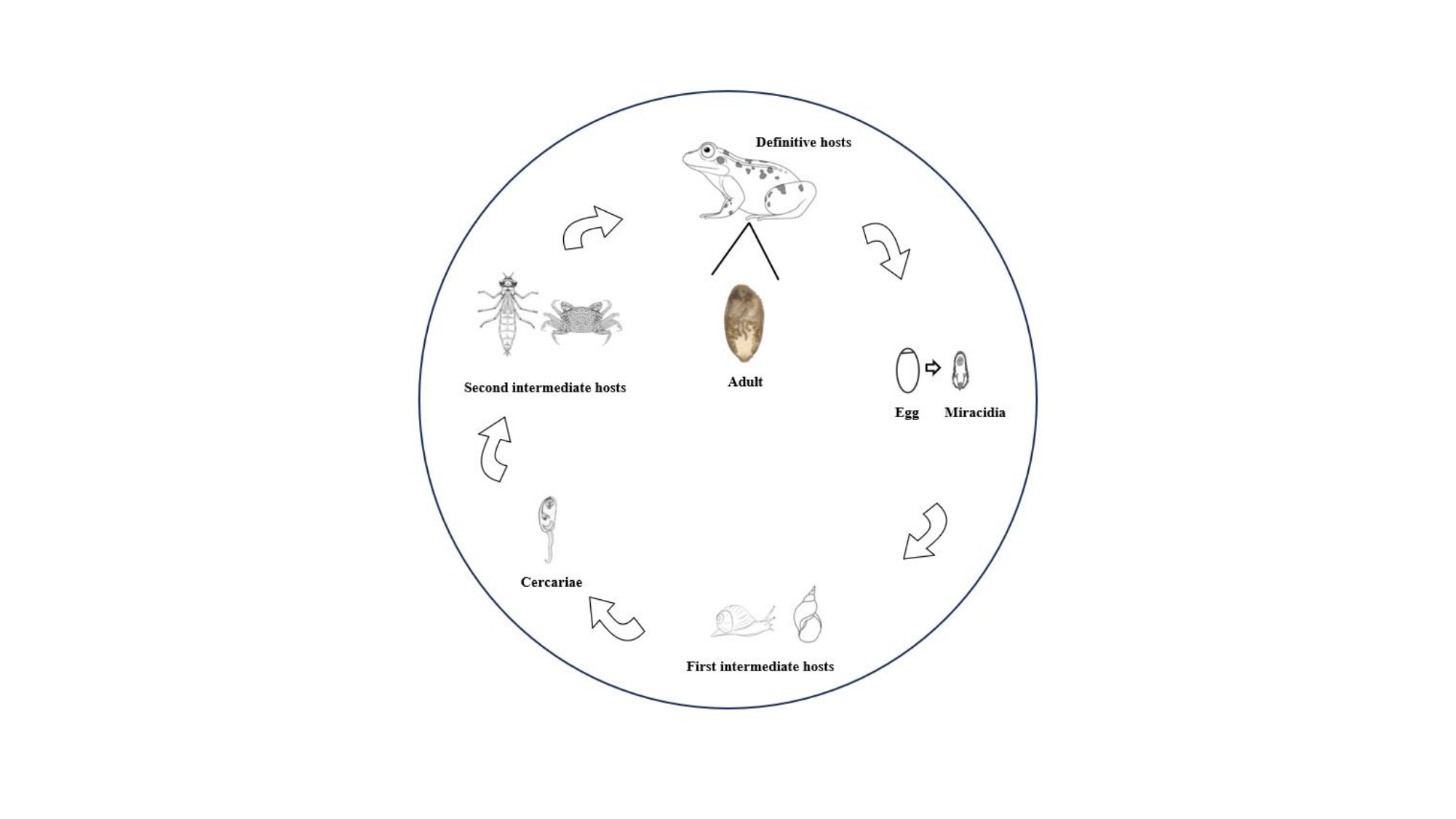

Supplement: Supplementary file 1 — Figure S1. The life cycle of Pleurogenoides trematodes. [file ECE3-14-e70430-s003.pptx]

## Slide 1
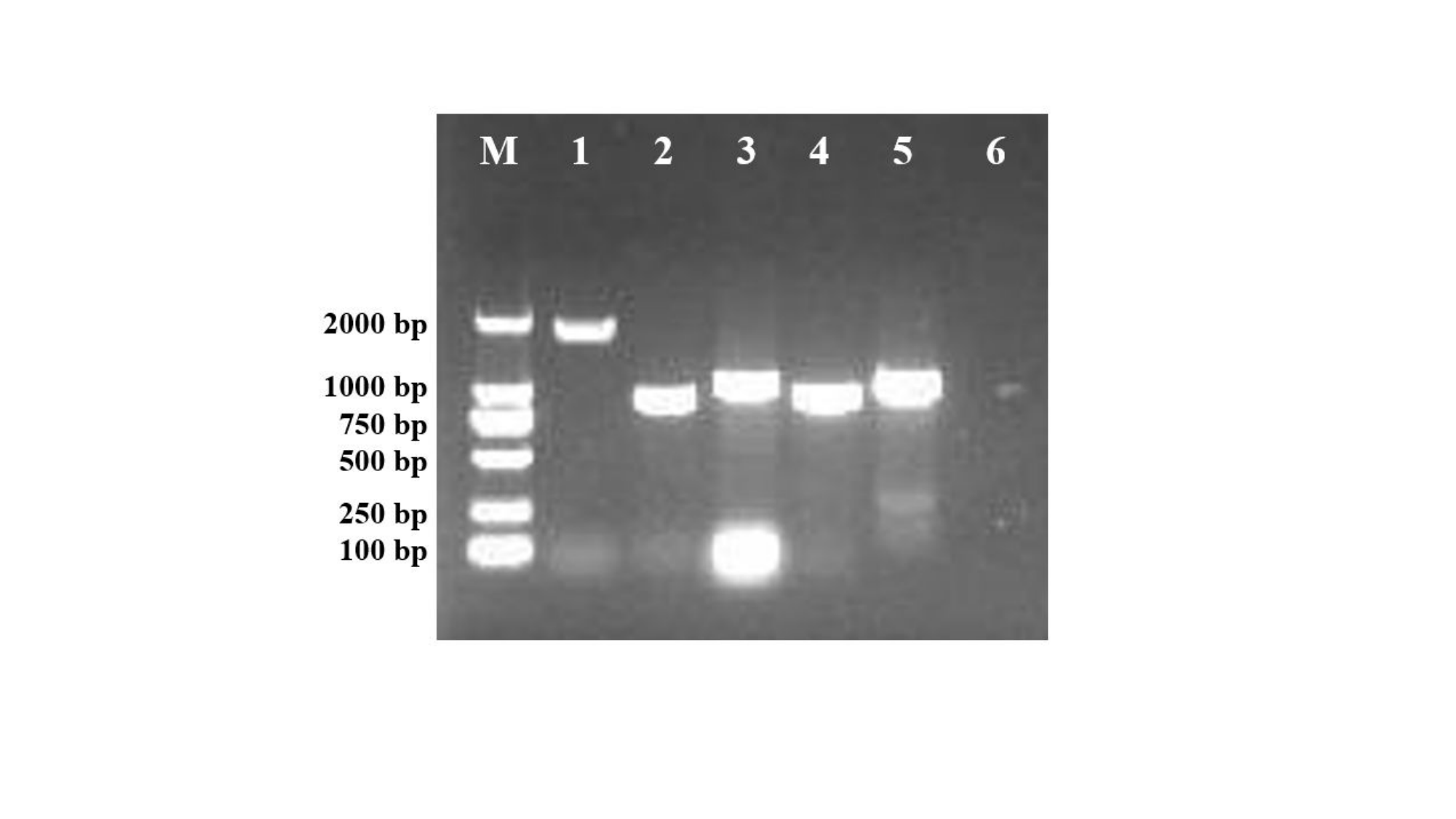

Supplement: Supplementary file 2 — Figure S2. PCR amplicons from the mitochondrial genome of Pleurogenoides japonicus. M: DL 2000 DNA marker, 1: Partial of nad5, 2: Partial of atp6, 3: Partial of nad3, 4: Partial of SNCR, 5: cox2; 6: Negative control. [file ECE3-14-e70430-s007.pptx]

## Slide 1
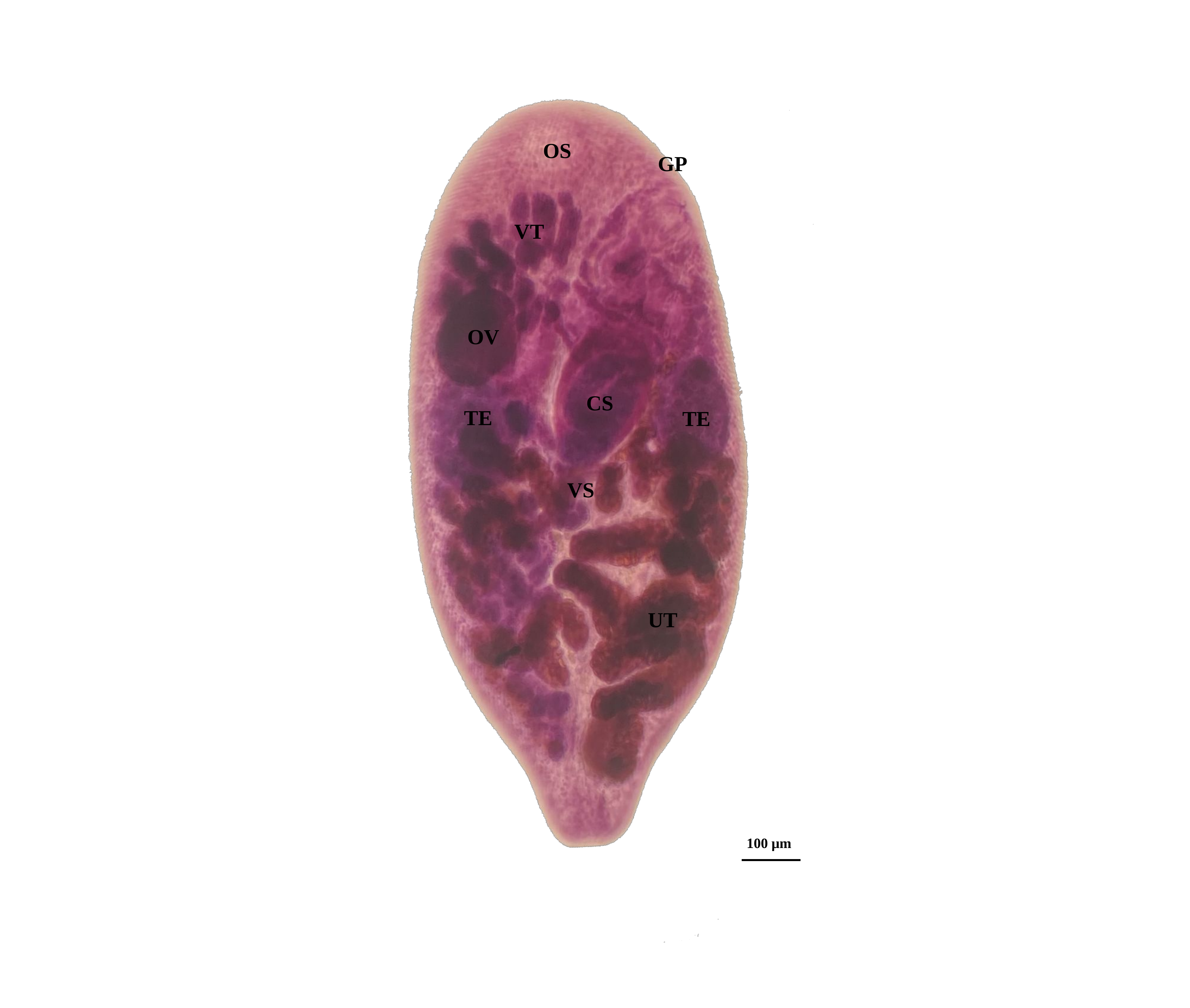

OS
GP
VT
OV
CS
TE
TE
VS
UT
100 μm

Supplement: Supplementary file 3 — Figure S3. Morphological characteristics of Pleurogenoides japonicus adult. OS, oral sucker; VS, ventral sucker; OV, ovary; TE, testes; VT, vitellaria; UT, uterus; CS, cirrus sac; GP, genital pore. [file ECE3-14-e70430-s005.pptx]

## Slide 1
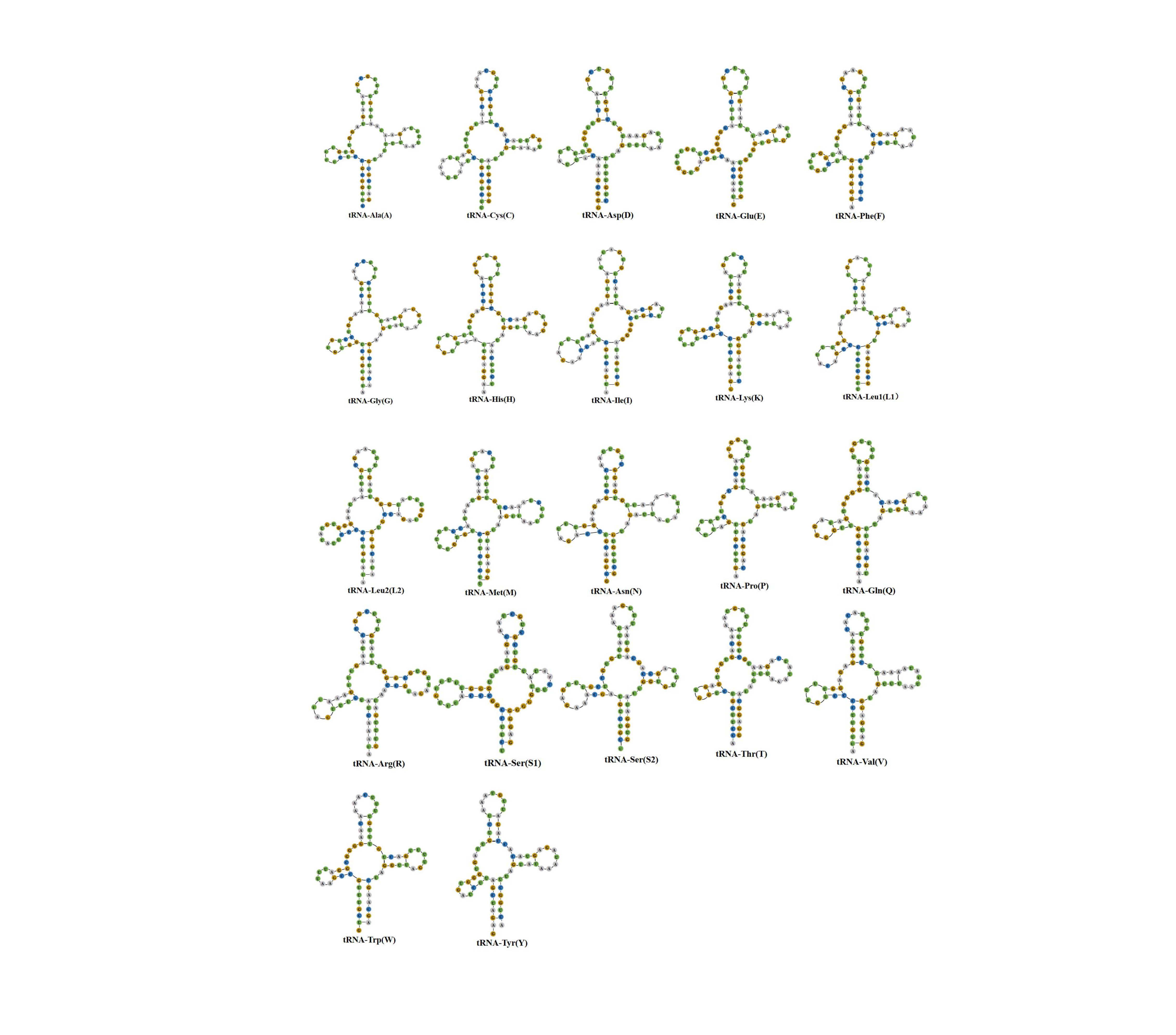

Supplement: Supplementary file 4 — Figure S4. Predicted structure model of 22 tRNAs from the mitochondrial genome of Pleurogenoides japonicus. [file ECE3-14-e70430-s004.pptx]
