# Supplementary material for: Characterization of the Complete Mitochondrial Genome of Pleurogenoides japonicus (Digenea, Pleurogenidae): Comparison With the Members of Microphalloidea and Phylogenetic Implications
Source: Ecol Evol. 2024 Oct 16;14(10):e70430. doi: 10.1002/ece3.70430 (PMC11483596; doi:10.1002/ece3.70430)
Supplement: Supplementary file 5 — Table S1. The nuclear large subunit ribosomal DNA sequences information of superfamily Microphalloidea used in phylogenetic analysis. [file ECE3-14-e70430-s009.docx]

**Table S1.** The nuclear large subunit ribosomal DNA sequences information of superfamily Microphalloidea used in phylogenetic analysis.

| Superfamily | Family | Species | GenBank Number |
| --- | --- | --- | --- |
| Microphalloidea | Cortrematidae | *Cortrema magnicaudata* | KJ700420 |
|  | Lecithodendriidae | *Macyella postgonoporus* | KY752115 |
|  |  | *Leyogonimus polyoon* | KY752116 |
|  | Pleurogenidae | *Allassogonoporus amphoraeformis* | AY220620 |
|  |  | *Parabascus duboisi* | AY220618 |
|  |  | *Parabascus joannae* | AY220619 |
|  |  | *Parabascus semisquamosus* | AF151923 |
|  |  | *Brandesia turgida* | AY220622 |
|  |  | *Prosotocus confusus* | AY220623 |
|  |  | *Candidotrema loossi* | AY220621 |
|  |  | *Pleurogenes claviger* | AF151925 |
|  |  | *Pleurogenoides medians* | AF433670 |
|  |  | *Pleurogenoides japonicus* | PQ285814 |
|  | Prosthogonimidae | *Prosthogonimus cuneatus* | AY220634 |
|  |  | *Prosthogonimus ovatus* | AF151928 |
|  | Microphallidae | *Maritrema arenaria* | AY220629 |
|  |  | *Maritrema prosthometra* | AY220631 |
|  |  | *Maritrema oocysta* | AY220630 |
|  |  | *Maritrema neomi* | AF151927 |
|  |  | *Maritrema brevisacciferum* | KT355819 |
|  |  | *Microphallus similis* | HM584139 |
|  |  | *Microphallus triangulatus* | HM584139 |
|  |  | *Microphallus basodactylophallus* | AY220628 |
|  |  | *Microphallus abortivus* | AY220626 |
|  |  | *Microphallus primas* | AY220627 |
|  | Lecithodendriidae | *Paralecithodendrium chilostomum* | AF151920 |
|  |  | *Paralecithodendrium longiforme* | AF151921 |
|  |  | *Lecithodendrium linstowi* | AF151919 |
|  |  | *Pycnoporus megacotyle* | AF151917 |
|  |  | *Pycnoporus heteroporus* | AF151918 |
| Gorgoderoidea | Dicrocoeliidae | *Dicrocoelium dendriticum* | DQ379986 |
