# Supplementary material for: Characterization of the Complete Mitochondrial Genome of Pleurogenoides japonicus (Digenea, Pleurogenidae): Comparison With the Members of Microphalloidea and Phylogenetic Implications
Source: Ecol Evol. 2024 Oct 16;14(10):e70430. doi: 10.1002/ece3.70430 (PMC11483596; doi:10.1002/ece3.70430)
Supplement: Supplementary file 6 — Table S2. Four pairs of primers used for the complete mitochondrial genome assembly validation of Pleurogenoides japonicus. [file ECE3-14-e70430-s002.docx]

**TABLE S1.** Four pairs of primers used for the complete mitochondrial genome assembly validation of *Pleurogenoides japonicus*.

| **Regions** | **Primers (5' to 3')** | **Locations** | **Sizes (bp)** |
| --- | --- | --- | --- |
| *nad*5 | F: TTTGATAACCGTAGATTGTGCG | 12477-14242 | ~1700 |
|  | R: CATTATACTTCCGATGTTACA |  |  |
| *atp*6 | F: TTTGGGCTGCTTACTGGTTCGT | 3257-4140 | ~880 |
|  | R: CCCCCAATAGGATACCCGCCAT |  |  |
| *nad3* | F: CTGGTTAGTGGGCTTAGGACGG | 5993-7039 | ~1040 |
|  | R: ACATAACCCCGCTCTATCTCCA |  |  |
| SNCR | F: GTCGCTGCTAACGATGATTTGG | 7084-7909 | ~820 |
|  | R: AAGTTCAACCGACACCAGAGCC |  |  |
| *cox*2 | F: TACACACCGCCCGTCACTCAC | 10958-11959 | ~1000 |
|  | R: AACCTACACCAACAACCGAAAA |  |  |
