# Supplementary material for: Characterization of the Complete Mitochondrial Genome of Pleurogenoides japonicus (Digenea, Pleurogenidae): Comparison With the Members of Microphalloidea and Phylogenetic Implications
Source: Ecol Evol. 2024 Oct 16;14(10):e70430. doi: 10.1002/ece3.70430 (PMC11483596; doi:10.1002/ece3.70430)
Supplement: Supplementary file 7 — Table S3. The complete mitochondrial genome information of trematodes used in phylogenetic analysis. [file ECE3-14-e70430-s006.docx]

**TABLE S2.** The complete mitochondrial genome information of trematodes used in phylogenetic analysis.

| Suborder | Superfamily | Family | Species | GenBank Number |
| --- | --- | --- | --- | --- |
| Pronocephalata | Paramphistomoidea | Diplodiscidae | *Diplodiscus japonicus* | OL961442 |
|  |  |  | *Diplodiscus mehari* | OL961441 |
|  |  | Paramphistomidae | *Paramphistomum cervi* | NC_023095 |
|  |  |  | *Paramphistomum leydeni* | KP341657 |
|  |  | Gastrothylacidae | *Fischoederius cobboldi* | NC_030529 |
|  |  |  | *Fischoederius elongatus* | NC_028001 |
|  | Pronocephaloidea | Notocotylidae | *Notocotylus intestinalis* | NC_059797 |
|  |  |  | *Ogmocotyle ailuri* | OP414758 |
| Xiphidiata | Troglotrematoidea | Paragonimidae | *Paragonimus westermani* | MN412706 |
|  |  |  | *Paragonimus ohirai* | NC_032032 |
|  |  |  | *Paragonimus heterotremus* | NC_039430 |
|  | Microphalloidea | Prosthogonimidae | *Prosthogonimus pellucidus* | MZ169556 |
|  |  |  | *Prosthogonimus cuneatus* | NC_050918 |
|  |  | Pleurogenidae | *Pleurogenoides japonicus* | OR900118 |
|  |  | Eucotylidae | *Tamerlania zarudnyi* | MW334947 |
|  | Plagiorchioidea | Plagiorchiidae | *Glypthelmins quieta* | MZ099629 |
|  |  |  | *Plagiorchis maculosus* | NC_042482 |
|  |  |  | *Plagiorchis elegans* | NC_071963 |
|  |  |  | *Plagiorchis multiglandularis* | NC_072063 |
|  | Gorgoderoidea | Dicrocoeliidae | *Dicrocoelium chinensis* | NC_025279 |
|  |  |  | *Dicrocoelium dendriticum* | NC_025280 |
|  |  |  | *Eurytrema pancreaticum* | KP241855 |
|  |  |  | *Lyperosomum longicauda* | NC_048467 |
|  | Allocreadioidea | Brachycladiidae | *Brachycladium goliath* | KR703278 |
| Echinostomata | Echinostomatoidea | Cyclocoelidae | *Tracheophilus cymbius* | NC_044135 |
|  |  | Fasciolidae | *Fascioloides magna* | KU060148 |
|  |  |  | *Fasciola hepatica* | AF216697 |
|  |  |  | *Fasciola gigantica* | MH621335 |
|  |  | Echinochasmidae | *Echinochasmus japonicus* | NC_030518 |
|  |  |  | *Echinostoma hortense* | KR062182 |
| Opisthorchiata | Opisthorchioidea | Opisthorchiidae | *Clonorchis sinensis* | MT607652 |
|  |  |  | *Opisthorchis felineus* | NC_011127 |
|  |  |  | *Opisthorchis viverrine* | JF739555 |
|  |  |  | *Metorchis orientalis* | NC_028008 |
|  |  | Heterophyidae | *Haplorchis taichui* | MG972809 |
|  |  |  | *Metagonimus yokogawai* | NC_023249 |
| Strigeidida | Schistosomatoidea | Schistosomatidae | *Schistosoma mansoni* | NC_002545 |
