# Supplementary material for: Characterization of the Complete Mitochondrial Genome of Pleurogenoides japonicus (Digenea, Pleurogenidae): Comparison With the Members of Microphalloidea and Phylogenetic Implications
Source: Ecol Evol. 2024 Oct 16;14(10):e70430. doi: 10.1002/ece3.70430 (PMC11483596; doi:10.1002/ece3.70430)
Supplement: Supplementary file 8 — Table S4. The A + T contents of mitochondrial genome in superfamily Microphalloidea trematodes. [file ECE3-14-e70430-s001.docx]

**TABLE S3.** The A + T contents of mitochondrial genome in superfamily Microphalloidea trematodes.

| Gene | *Pleurogenoides japonicus* | | *Prosthogonimus cuneatus* | | *Prosthogonimus pellucidus* | | *Tamerlania zarudnyi* | |
| --- | --- | --- | --- | --- | --- | --- | --- | --- |
|  | A + T contents (%) | G + C contents (%) | A + T contents (%) | G + C contents (%) | A + T contents (%) | G + C contents (%) | A + T contents (%) | G + C contents (%) |
| *cox*3 | 66.06 | 33.94 | 67.28 | 32.72 | 66.51 | 33.49 | 63.75 | 36.25 |
| *cyt*b | 63.56 | 36.44 | 64.35 | 35.65 | 65.69 | 34.31 | 61.81 | 38.19 |
| *nad*4L | 68.2 | 31.8 | 65.97 | 34.03 | 68.06 | 31.94 | 61.11 | 38.89 |
| *nad*4 | 64.48 | 35.52 | 66.67 | 33.33 | 68.02 | 31.98 | 60.67 | 39.33 |
| *atp*6 | 64.56 | 35.44 | 67.02 | 32.98 | 68.26 | 31.74 | 63.87 | 36.13 |
| *nad*2 | 63.66 | 36.34 | 67.24 | 32.76 | 66.32 | 33.68 | 62.07 | 37.93 |
| *nad*1 | 63.99 | 36.01 | 64.11 | 35.89 | 68.78 | 31.22 | 62.43 | 37.57 |
| *nad*3 | 70.34 | 29.66 | 69.47 | 30.53 | 70.31 | 29.69 | 64.15 | 35.85 |
| *cox*1 | 62.26 | 37.74 | 63.74 | 36.26 | 64.39 | 35.61 | 59.61 | 40.39 |
| *cox*2 | 61.31 | 38.69 | 61.11 | 38.89 | 63.47 | 36.53 | 59.97 | 40.03 |
| *nad*6 | 64.68 | 35.32 | 67.11 | 32.89 | 67.32 | 32.68 | 65.33 | 34.67 |
| *nad*5 | 64.86 | 35.14 | 65.29 | 34.71 | 68.36 | 31.64 | 58.46 | 41.54 |
| 12PCGS | 64.21 | 35.79 | 65.39 | 34.61 | 66.86 | 33.14 | 61.15 | 38.85 |
| *rrn*L | 62.27 | 37.73 | 63.05 | 636.95 | 62.4 | 37.6 | 58.04 | 41.96 |
| *rrn*S | 61.41 | 38.59 | 64.59 | 35.41 | 63.03 | 36.97 | 56.07 | 43.93 |
| tRNAs | 59.92 | 40.08 | 58.82 | 41.18 | 59.57 | 40.43 | 57.04 | 42.96 |
| overall | 63.75 | 36.25 | 64.47 | 35.53 | 65.34 | 34.65 | 60.58 | 39.42 |
