# Supplementary material for: Characterization of the Complete Mitochondrial Genome of Pleurogenoides japonicus (Digenea, Pleurogenidae): Comparison With the Members of Microphalloidea and Phylogenetic Implications
Source: Ecol Evol. 2024 Oct 16;14(10):e70430. doi: 10.1002/ece3.70430 (PMC11483596; doi:10.1002/ece3.70430)
Supplement: Supplementary file 9 — Table S5. The A + T and G + C skew of mitochondrial genome in superfamily Microphalloidea trematodes. [file ECE3-14-e70430-s008.docx]

**TABLE S4.** The A + T and G + C skew of mitochondrial genome in superfamily Microphalloidea trematodes.

| Genes | *Pleurogenoides japonicus* | | *Prosthogonimus cuneatus* | | *Prosthogonimus pellucidus* | | *Tamerlania zarudnyi* | |
| --- | --- | --- | --- | --- | --- | --- | --- | --- |
|  | AT-skew | GC-skew | AT-skew | GC-skew | AT-skew | GC-skew | AT-skew | GC-skew |
| *cox*3 | -0.505 | 0.357 | -0.595 | 0.449 | -0.600 | 0.388 | -0.566 | 0.432 |
| *cyt*b | -0.420 | 0.436 | -0.554 | 0.470 | -0.513 | 0.472 | -0.497 | 0.445 |
| *nad*4L | -0.472 | 0.542 | -0.558 | 0.531 | -0.541 | 0.543 | -0.564 | 0.524 |
| *nad*4 | -0.532 | 0.542 | -0.623 | 0.531 | -0.527 | 0.543 | -0.512 | 0.524 |
| *atp*6 | -0.614 | 0.475 | -0.612 | 0.449 | -0.501 | 0.363 | -0.487 | 0.258 |
| *nad*2 | -0.549 | 0.439 | -0.612 | 0.514 | -0.551 | 0.445 | -0.515 | 0.285 |
| *nad*1 | -0.516 | 0.486 | -0.563 | 0.492 | -0.499 | 0.530 | -0.536 | 0.513 |
| *nad*3 | -0.566 | 0.600 | -0.548 | 0.541 | -0.530 | 0.660 | -0.572 | 0.547 |
| *cox*1 | -0.448 | 0.378 | -0.447 | 0.388 | -0.397 | 0.444 | -0.411 | 0.308 |
| *cox*2 | -0.306 | 0.290 | -0.416 | 0.394 | -0.379 | 0.392 | -0.441 | 0.414 |
| *nad*6 | -0.597 | 0.538 | -0.667 | 0.520 | -0.596 | 0.557 | -0.510 | 0.244 |
| *nad*5 | -0.522 | 0.518 | -0.612 | 0.592 | -0.498 | 0.568 | -0.518 | 0.418 |
| 12PCGS | -0.498 | 0.440 | -0.564 | 0.486 | -0.499 | 0.482 | -0.496 | 0.388 |
| *rrn*L | -0.281 | 0.339 | -0.290 | 0.257 | -0.297 | 0.319 | -0.235 | 0.375 |
| *rrn*S | -0.202 | 0.284 | -0.169 | 0.313 | -0.139 | 0.299 | -0.040 | 0.231 |
| tRNAs | -0.230 | 0.356 | -0.283 | 0.357 | -0.271 | 0.361 | -0.217 | 0.351 |
| overall | -0.435 | 0.407 | -0.482 | 0.449 | -0.433 | 0.452 | -0.384 | 0.380 |
